# Health-related word recognition and pronunciation by patients in Gauteng, South Africa in English and native languages

**DOI:** 10.4102/phcfm.v16i1.4492

**Published:** 2024-07-22

**Authors:** Boitumelo Ditshwane, Zelda Janse van Rensburg, Wanda Jacobs

**Affiliations:** 1 Department of Nursing, Faculty of Health Sciences, University of Johannesburg, Johannesburg, South Africa

**Keywords:** word recognition, pronunciation, primary healthcare, native language, English, health literacy

## Abstract

**Background:**

Low health literacy has been found to affect people’s ability to take care of their own health and follow the principles of disease prevention. Incomprehension of health education and healthcare instructions may lead to poor health outcomes.

**Aim:**

The aim of the study was to describe and compare a sample of primary healthcare patient’s ability to recognise and pronounce health-related words in English and in his or her native language.

**Setting:**

The study was conducted in 12 primary healthcare (PHC) clinics in Gauteng, South Africa.

**Methods:**

A prospective, quantitative, comparative research design using a survey method was used to assess the ability to recognise and pronounce health-related words of 401 respondents using the REALM-R (SA) tool.

**Results:**

Most respondents were 18–29 years (32%) and 30–49 years (53%) old. More than half (54%) of the respondents have completed grade 12 schooling. Adequate English health-related word recognition and pronunciation levels were at 19.5%, while native health-related word recognition and pronunciation levels were far better, ranging between 55.6% and 97.0%.

**Conclusion:**

Respondents showed better word recognition and pronunciation of the health-related words in their native language than in English. Providing health information in the patient’s native language and on their level of understanding may therefore improve patient health outcomes.

**Contribution:**

The study is the first of its kind to determine word recognition and pronunciation of health-related words in English and a native language of South African PHC patients. Knowing this may assist healthcare professionals to give health education and instructions on the patient’s level of understanding.

## Introduction

Literacy is described as an individual’s competency to read, write, and speak a language with understanding and show the ability to solve problems to sufficiently function in the working environment and in society, achieve goals, and develop knowledge and individual potential.^1^ Globally the literacy rate for those 15 years and older is currently at 86.3%.^1^ This leaves 13.7% of the world illiterate. Developed nations such as countries in Asia, Canada, Russia and Poland have literacy rates of 96% and higher. Developing countries such as Chad, South Sudan, Somalia and Afghanistan have literacy rates as low as 27% – 45%.^1^ According to the United Nations Educational, Scientific and Cultural Organization (UNESCO) report, sub-Saharan Africa has one of the lowest adult literacy rates in the world with a 61% average literacy rate.^2^ According to the National household survey conducted in 2020, South Africa has a literacy rate of 87%, leaving 13% of the country functionally illiterate.^3^ Low literacy is defined as the inability of an individual to read and write.^3^ Low literacy and low health literacy (HL) are closely related.^4,5,6,7^

Health literacy is defined as competencies and skills that individuals need to find, comprehend, evaluate, and use to make knowledgeable choices to improve their health and well-being.^8,9,10^ According to the Institute of Medicine (IoM) model of HL, HL has four major components, namely cultural and conceptual knowledge, print HL (reading and writing), oral HL (listening and speaking), and numeracy.^11^ The IoM model of HL has a strong focus on the required skills for people to obtain, process, and apply information for the purpose of healthcare.^11^ An important component of HL is basic reading skills. Basic reading skills are necessary to function in everyday situations in healthcare, for instance reading prescription labels and health education materials.^11^ Reading also forms part of the ability to process and use health information in various formats in relation to health and healthcare.^11.^

Low HL has been found to affect people’s ability to take care of their own health and follow the principles of disease prevention and this as a result contributes to the spread of disease and health disparities.^1,12,13^ It has also been reported that some patients may feel embarrassed to admit that they have low literacy and as a result do not comprehend and therefore do no follow health education and healthcare instructions as they should.^9,14^ As a result of incomprehension of health education and healthcare instructions, patients’ ability to make correct choices and decisions regarding their health and well-being are compromised.^15^ These incorrect choices in turn lead to several adverse health outcomes because of non-compliance to treatment and treatment failures resulting in an increased morbidity and mortality rates.^1,12^

The incomprehension of health education and healthcare instructions because of low HL is often because of information given at a level that is above the patient’s level of understanding^6,12,16^ or in a language that is not well understood by the patient. Patients with low HL often find it difficult to follow health education and healthcare instructions such as how to take their medications and how to apply disease preventative measures.^1,6,17,18,19^ As basic reading is an important component of HL, patients with low HL may often lack the ability to read and to understand written healthcare instructions and health education materials.^12^

In South Africa, Primary Health Care (PHC) clinics are the first entry point for accessing healthcare services for the people in the community.^20,21^ It is therefore important for patients at PHC level of care to understand the health education and healthcare instructions given in either written or oral form, to avoid adverse health outcomes and the need for referral to higher levels of care.^22,23.^

Health education in written and oral form is the cornerstone of health promotion, disease prevention and improved health outcomes, especially at PHC level.^24,25,26^ Health education materials such as posters, pamphlets, and booklets, are mostly written in English.^15,28^ Oral health education is also often given in English as English is the dominant language for communication in the public sphere in South Africa.^12^ The lack of availability of health education materials in all 11 official South African languages may be a challenge for patients with low HL, as they might not be able to read and understand the information written in a language that they are less familiar or unfamiliar with.^12,27,28^ People in the community who are accessing PHC facilities also come from different socio-demographic backgrounds. This implies that some patients lack education while others have at least completed certain levels of education. As a result, some patients often find it challenging to understand health education and healthcare instructions if not given at their level of understanding or in a language that they are familiar with.^6,12^ A South African study found that patients’ level of understanding is four school grade levels below the highest level of schooling completed.^12^ Patients might also understand health education and healthcare instructions better when it is given in their native language or a language that they more frequently read, write, and speak.^29,30,31^

It is paramount for PHC providers to determine the HL levels of PHC patients to ensure that health education and healthcare instructions are well understood by patients in the PHC facilities.^1,12.^ One component of HL that should be tested includes the patient’s ability to recognise and pronounce health-related words that are frequently used in the PHC setting.^12^ If patients understand the health education and healthcare instructions given to them by PHC providers, patients might adhere to and comply well with treatment and this would assist in reducing rates of defaulting on treatment for conditions such as diabetes, hypertension, Human Immunodeficiency Virus./Acquired Immune Deficiency Syndrome (HIV/AIDS), and tuberculosis (TB), and improving overall health outcomes.^32,33^

The Rapid Estimate of Adult Literacy in Medicine–Revised (South Africa) or the REALM-R (SA) is the tool currently available to determine one component of HL, which is, word recognition and pronunciation of health-related words commonly used in the PHC context.^12,34,35^ The tool was only validated in the English language. The availability of this tool in only English was found challenging, as South Africa has 11 official languages for communication.^34,36^ Although English remains the dominant language in the country, many patients do not understand or have limited understanding of English in either written or oral form. Recognising and pronouncing health-related words in either English or in a native South African language therefore only forms part of the one component of HL; however, this remains the foundation of HL.

Assessing PHC patients’ health-related word recognition and pronunciation as a component of HL in their native language may give a more accurate indication of the patient’s ability to read and comprehend health care instructions and health education. If this can be determined, health education can be tailored to the needs of the patient and given to patients on their level of understanding that may increase PHC patients’ ability to understand and execute health education and healthcare instructions. In turn, patients may also be more likely to manage their medical conditions more effectively and to adhere to prescribed medication and improve their health outcomes when they understand what needs to be done to achieve this.^9,12^ These improved health outcomes may also be the right step in achieving the Sustainable Developmental Goal (SDG) 3: ‘Ensuring healthy lives and well-being for all’.^36^ This article gives a comparison between the health-related word recognition and pronunciation levels of PHC patients in English and in the patient’s native language.

## Research methods and design

### Study design and setting

A prospective, quantitative, comparative research design with a survey method was used to determine and compare the health-related word recognition and pronunciation levels of PHC patients attending 12 PHC clinics in Gauteng, South Africa. The data were collected onsite at the 12 PHC clinics that were selected by the researcher across Gauteng who catered for PHC patients who spoke the nine native languages using a stratified sampling method to give a representative sample. The clinics were located as follows: three clinics in the City of Johannesburg and Tshwane, two from Sedibeng, two from the West Rand district, and two from the Ekurhuleni. All the clinics had a diversity of people from different races, cultural and linguistic backgrounds. The languages spoken in Gauteng includes IsiZulu (25.1%), English (16.6%), IsiXhosa (12.8%), Afrikaans (9.7%), Sepedi (9.7%), Setswana (9.4%), Sesotho (7.8%), Swati (2.6%), XiTsonga (2.4%), Tshivenda (2.2%), and isiNdebele (1.3%). The isiNdebele language was excluded in this study because of a small percentage of Ndebele speaking people in Gauteng. A descriptive design was used to describe and summarise the collected data.

### Study population and sampling strategy

Primary Health Care patients, 18 years and older were included in the study using a stratified sampling method. Stratified sampling allowed the researcher to ensure that different groups within a population were represented proportionally in the sample. Respondents from different areas in Gauteng and speaking one other native language besides English (excluding isiNdebele) needed to be included in the study for a representative sample. This method allowed the researchers to assess the health-related word recognition and pronunciation levels of 401 PHC patients who met the inclusion criteria and who self-declared the ability to read and speak both English and another one of the 11 official South African native language. A predetermined sample size of 400 respondents was chosen with the assistance of a statistician to meet the aims and objectives of the study. A minimum of 33 patients per PHC clinic were targeted allowing representatives of all nine native languages, with a 95% confidence interval with an error margin of 5%. However, the final number of respondents was 401. Primary Health Care patients who were severely ill or presented as emotionally or mentally disabled or challenged were not approached to participate in the study.

### Data collection and instrument

Prospective respondents were approached by the researcher on the day they attended the clinic, while they were waiting to be consulted. The purpose of the study was explained to the patients by the researcher. Those who were willing to participate and met the inclusion criteria were included in the study. Patients were taken from the consultation waiting line. Their place was kept until they returned. The patients were taken to a private consultation room where the REALM-R (SA) assessments took place. The patients returned to their place in line after the assessments. If the patient chose to do the assessment after their consultation, they were told to wait outside the private consultation room and the researcher will take them to be assessed.

### The Rapid Estimate of Adult Literacy in Medicine–Revised (South Africa)

The original REALM-R was developed in the United States of America as a HL assessment tool based on word recognition and pronunciation of health-related words.^3,12^ The REALM-R was locally adapted for use in South Africa by Wassermann et al.^34^ in a pilot study to create three different REALM-R (SA) tools. These tools were further validated by Janse van Rensburg^12^ to determine which one of them would be most suitable to assess the health-related word recognition and pronunciation levels of South African PHC patients. The final validated tool was named the REALM-R (SA) by Janse van Rensburg.^12^ The REALM-R (SA) was however only validated in English.^12^

With the REALM-R (SA), PHC patients are asked to recognise health-related words commonly used in the PHC setting, by reading and then pronouncing the words. Pronunciation was scored on the ability of the patient to correctly pronounce the words according to a phonetic pronunciation key.^12^ Although the original REALM-R (SA) was a paper-based tool, in this study the REALM-R was converted into a Google Form and used for assessment purposes via a tablet or smart phone by the researcher. The 11 English words included were: *Food, Germs, Pain, Treatment, Condom, Transmission, Vomiting, Prevention, Hypertension, Tuberculosis, and Osteoporosis.* For both the English REALM-R (SA) and the translated REALM-R (SA) in the native South African languages, the first three words were not scored and only used to put the patient at ease. A final score for correct word recognition and pronunciation of the word was allocated out of eight. The scoring was performed sequentially, and the words range from easy to more difficult to pronounce. If the patient hesitated for more than 5 s, the researcher would say ‘pass’ and the respondent was given the opportunity to read the next word. The missed word would be scored as a ‘0’. The respondents received an opportunity to self-correct the mispronounced or unpronounced word before the total score was calculated. The Google Forms automatically calculated a score out of eight and captured the score on a Microsoft Excel spreadsheet. Scores were indicated as follows^12^: Five or less out of eight was considered as low health-related word recognition and pronunciation, six to seven out of eight as moderate health-related word recognition and pronunciation, and eight out of eight as an adequate health-related word recognition and pronunciation level.

In addition to assessing the health-related word recognition and pronunciation of the respondents in English, the REALM-R (SA) was translated into eight other South African native languages by a linguistic expert (see Table 1). The health-related words on the translated REALM-R (SA) tools were also read and recorded by the linguistic expert to ensure that the researcher could replay the words and hear the correct pronunciation of the words. The respondents received the opportunity to firstly be assessed in English and secondly to chose one native South African language to also be assessed. The languages included: IsiZulu, English, IsiXhosa, Afrikaans, Sepedi, Setswana, Sesotho, Swati, XiTsonga, Tshivenda.

**TABLE 1:** Translated medical words on the Rapid Estimate of Adult Literacy in Medicine–Revised (South Africa).

Language	Translated medical words on the REALM-R (SA)
Afrikaans	*Behandeling, Kondoom, Oordraag, Braking, Voorkoming, Hipertensie, Tuberkulose and Osteoporose*
Sepedi	*Kalafo, Kgotlopo, Phetiṧetṧo, Go hlatsa, Thibela, Kgatelelo ya Madi, TB and Go fokola ga Marapo*
Sesotho	*Pheko, Kgohlopo, Tshwaetsano, Ho Hlatsa, ho Thibela, Kgatello e hodimo ya Madi, Lefuba and Bofokodi ba Masapo*
XiTsonga	*Vutshunguri, Khondomu, Ntluleto, Ku hlanta, Nsivelo, High Blood, Vubabyi bya Rifuva and Ku tsana ka Marambu*
Tshivenda	*Dzilafho, Tshitsireledzi, U Pufkela, U tanza, U Thivhela, Mutsiko wa Malofha, Vhuladze ha Mafhafhu and Vhulwadze ha Marambo*
IsiXhosa	*Unyango, Idyasi Yomkhwenyane, Usulelo, Uk’gabha, Ithintelo, Unxinzelelo Lwegazi, Isifo Sephepha and Kwamathambo*
IsiZulu	*Ezokwelapha, Ijazi Likamkhwenyana, Ukwesulela/Ukuthathelana, Ukuphalaza/Ukuhlanza, Ukuvimbela, Umfutho Wegazi OpheisiZulu, Isifo Sofuba and Isifo Samathambo*
Swati	*Tindlela Tekulapha, Lijazi Lemkhwenyana, Kwendluliswa, Kuphalaza, Kugwema, Umfutho o Phetulu, TB and Kugula KweMatsambo*
Setswana	*Kalafi, Mosomelwana, Tshwaetso, Go tlhatsa, Thibelo, Kgatelelo ya Madi, Sefuba se Setona and Koafalo ya Marapo*

*Source:* Translated from the REALM-R (SA) by Janse van Rensburg Z. Levels of health literacy and English comprehension in patients presenting to South African primary healthcare facilities. Afr J Prim Health Care Fam Med. 2020;12(1):a2047. https://doi.org/10.4102/phcfm.v12i1.2047

REALM-R (SA), Rapid Estimate of Adult Literacy in Medicine–Revised (South Africa).

The REALM-R (SA) tools were pilot tested with 10 respondents in one PHC clinic to evaluate the validity and reliability of the tool and to test the data collection process. The results of the pilot test were not included in the data set. The English REALM-R (SA) tool has previously been validated in the South African health context by Janse van Rensburg^12^ and permission was granted for the tool to be translated into nine other languages as well as for the HL assessment in English.

### Data analysis

The data were automatically captured on a Microsoft Excel spreadsheet via Google Forms before it was sent to a statistician for data analysis. The Statistical Package for the Social Sciences IBM SPSS Statistics (Version 28), New York, United States was used to analyse the data and to make comparisons of the health-related word recognition and pronunciation in English and the South African native languages.

### Ethical considerations

Ethical approval to conduct the study was received from the Research Ethics Committee (REC-1897-2023) at the University of Johannesburg and the Gauteng Department of Health (GP_202302_057). Respondents gave written informed consent to participate in the study and had the right to withdraw from the study at any time without any negative consequences. The consent included the completion of the REALM-R (SA) tools on Google Forms. The assessments took place in a private room at the clinic to prevent psychological discomfort when the respondents were not able to pronounce a word. The respondents were put at ease by allowing them to read the first three words on the English and native language REALM-R (SA) tools without being scored. The researcher was sensitive to the respondent’s reading levels and did not attempt to create a feeling of embarrassment when the respondent was unable to pronounce a word correctly. The researcher simply stated, ‘would you like to try again, or move on to the next word’? Data were collected anonymously and are kept in a password protected folder for five years after publication of the results as per the standard operating procedure of the university under which the study was conducted.

## Results

### Demographics

The demographics of the respondents are indicated in Table 2. Most respondents were between the ages of 30–49 years (53%). Little more than half (54%) of the respondents completed high school (grade 12) and 42% did not complete high school. Most of the respondent’s native language were Sepedi (20.1%) or Setswana (20.1%) followed by IsiZulu (16.7%) and Sesotho (14.5%).

**TABLE 2:** Demographic data of the participants (*N* = 401).

Variable	Frequency	%
**Age in years**		
18–29	128	32
30–49	213	53
50–69	57	14
69–80	3	0.1
**Highest level of schooling completed**		
No schooling	1	0.1
Grade 12 completed	216	54
High school not completed	168	42
Primary school not completed	16	3.9
**Preferred native language**		
Afrikaans	18	4.5
Sepedi	79	19.7
Setswana	60	15.0
Sesotho	29	7.2
Swati	22	5.5
XiTsonga	35	8.7
Tshivenda	67	16.7
IsiXhosa	11	2.7
IsiZulu	80	20.0

**Total**	**401**	**100.0**

### English health-related word recognition and pronunciation

The eight words that the respondents were asked to read and pronounce for scoring were in the following order: *Food, Germs, Pain, Treatment, Condom, Transmission, Vomiting, Prevention, Hypertension, Tuberculosis, and Osteoporosis*. Majority of the respondents scored either 6/8 (20.4%) and 7/8 (42.1%) in the English health-related word recognition and pronunciation assessment (moderate) using the REALM-R (SA). Only 19.5% of the respondents showed an adequate health-related word recognition and pronunciation level in English (Table 3).

**TABLE 3:** Respondent’s English health-related word recognition and pronunciation levels (*N* = 401).

Score in English	Frequency	%	Level
1	5	1.2	Low
2	6	1.5	Low
3	8	2.0	Low
4	11	2.7	Low
5	42	10.5	Low
6	82	20.5	Moderate
7	169	42.1	Moderate
8	78	19.5	Adequate

**Total**	**401**	**100.0**	**-**

### Native South African languages chosen for health-related word recognition and pronunciation assessment

While the respondents indicated their native language they usually speak (Table 4), some chose a different language for the health-related word recognition and pronunciation assessment. Majority of the respondents chose Setswana (20%), Sepedi (19.7%), Sesotho (15%), and IsiZulu (16.7%).

**TABLE 4:** Native language chosen for health-related word recognition and pronunciation assessment (*N* = 401).

Native language chosen	Frequency	%
Afrikaans	18	4.5
Sepedi	79	19.7
Sesotho	60	15.0
XiTsonga	29	7.2
Tshivenda	22	5.5
IsiXhosa	35	8.7
IsiZulu	67	16.7
Swati	11	2.7
Setswana	80	20.0

Total	401	100.0

### English and native health-related word recognition and pronunciation levels comparison

The health-related word recognition and pronunciation levels in English and the health-related word recognition and pronunciation levels in the chosen native language were compared according to the score received out of eight (Table 5a–Table 5i).

**TABLE 5a:** English health-related and native South African language word recognition and pronunciation levels compared (*N* = 18).

Score	Word recognition and pronunciation levels in Afrikaans			Word recognition and pronunciation levels in English		
	Frequency	%	Level	Frequency	%	Level
4	1	5.6	Low	0	0	-
5	3	16.7	Low	0	0	-
6	2	11.1	Moderate	6	33.3	Moderate
7	2	11.1	Moderate	6	33.3	Moderate
8	10	55.6	Adequate	6	33.3	Adequate

**Total**	**18**	**100.0**	**-**	**18**	**100.0**	**-**

**TABLE 5b:** English health-related and native South African language word recognition and pronunciation levels compared (*N* = 79).

Score	Word recognition and pronunciation levels in Sepedi			Word recognition and pronunciation levels in English		
	Frequency	%	Level	Frequency	%	Level
1	1	1.3	Low	2	2.5	Low
2	1	1.3	Low	1	1.3	Low
4	0	0	-	2	2.5	Low
5	4	5.1	Low	5	6.3	Low
6	4	5.1	Moderate	17	21.5	Moderate
7	17	21.5	Moderate	41	51.9	Moderate
8	52	65.8	Adequate	11	13.9	Adequate

**Total**	**79**	**100.0**	**-**	**79**	**100.0**	**-**

**TABLE 5c:** English health-related and native South African language word recognition and pronunciation levels compared (*N* = 60).

Score	Word recognition and pronunciation levels in Sesotho			Word recognition and pronunciation levels in English		
	Frequency	%	Level	Frequency	%	Level
1	0	0	-	1	1.7	Low
2	1	1.7	Low	1	1.7	Low
3	1	1.7	Low	5	8.3	Low
4	1	1.7	Low	3	5.0	Low
5	0	0	Low	5	8.3	Low
6	1	1.7	Moderate	12	20.0	Moderate
7	2	3.3	Moderate	20	33.3	Moderate
8	54	90.0	Adequate	13	21.7	Adequate

**Total**	**60**	**100.0**	**-**	**60**	**100**	**-**

**TABLE 5d:** English health-related and native South African language word recognition and pronunciation levels compared (*N* = 29).

Score	Word recognition and pronunciation levels in XiTsonga			Word recognition and pronunciation levels in English		
	Frequency	%	Level	Frequency	%	Level
2	0	0	-	2	6.9	Low
5	2	6.9	Low	2	6.9	Low
6	0	-	-	5	17.2	Moderate
7	1	3.4	Moderate	18	62.1	Moderate
8	26	89.7	Adequate	2	6.9	Adequate

**Total**	**29**	**100.0**	**-**	**29**	**100.0**	**-**

**TABLE 5e:** English health-related and native South African language word recognition and pronunciation levels compared (*N* = 22).

Score	Word recognition and pronunciation levels in Tshivenda			Word recognition and pronunciation levels in English		
	Frequency	%	Level	Frequency	%	Level
4	0	0	-	1	4.5	Low
5	0	0	-	4	18.2	Low
6	0	0	-	3	13.6	Moderate
7	1	4.5	Moderate	11	50.0	Moderate
8	21	95.5	Adequate	3	13.6	Adequate

**Total**	**22**	**100.0**	**-**	**22**	**100.0**	**-**

**TABLE 5f:** English health-related and native South African language word recognition and pronunciation levels compared (*N* = 35).

Score	Word recognition and pronunciation levels in IsiXhosa			Word recognition and pronunciation levels in English		
	Frequency	%	Level	Frequency	%	Level
5	0	0	-	6	17.1	Low
6	0	0	-	7	20.0	Low
7	2	5.7	Moderate	12	34.3	Moderate
8	33	94.3	Adequate	10	28.6	Adequate

**Total**	**35**	**100.0**	**-**	**35**	**100.0**	**-**

**TABLE 5g:** English health-related and native South African language word recognition and pronunciation levels compared (*N* = 67).

Score	Word recognition and pronunciation levels in IsiZulu			Word recognition and pronunciation levels in English		
	Frequency	%	Level	Frequency	%	Level
2	1	1.5	Low	1	1.5	Low
3	0	0.0	-	1	1.5	Low
4	0	0.0	-	2	3.0	Low
5	0	0.0	-	11	16.4	Low
6	0	0.0	-	12	17.9	Moderate
7	1	1.5	Moderate	23	34.3	Moderate
8	65	97.0	Adequate	17	25.4	Adequate

**Total**	**67**	**100.0**	**-**	**67**	**100.0**	**-**

**TABLE 5h:** English health-related and native South African language word recognition and pronunciation levels compared (*N* = 11).

HL score	Word recognition and pronunciation levels in in Swati			Word recognition and pronunciation levels in English		
	Frequency	%	HL level	Frequency	%	HL level
3	1	9.1	Low	0	0	-
4	0	0.0	-	1	9.1	Low
5	0	0.0	-	2	18.2	Low
6	0	0.0	-	3	27.3	Moderate
7	1	9.1	Moderate	3	27.3	Moderate
8	9	81.8	Adequate	2	18.2	Adequate

**Total**	**11**	**100.0**	**-**	**11**	**100.0**	**-**

**TABLE 5i:** English health-related and native South African language word recognition and pronunciation levels compared (*N* = 80).

HL score	Word recognition and pronunciation levels in in Setswana			Word recognition and pronunciation levels in English		
	Frequency	%	HL level	Frequency	%	HL level
1	1	1.3	Low	2	2.5	Low
2	1	1.4	Low	1	1.3	Low
3	0	0.0	-	2	2.5	Low
4	2	2.5	Low	2	2.5	Low
5	2	2.5	Low	7	8.8	Low
6	6	7.5	Moderate	17	21.3	Moderate
7	13	16.3	Moderate	35	43.8	Moderate
8	55	68.8	Adequate	14	17.5	Adequate

**Total**	**80**	**100.0**	**-**	**80**	**100.0**	

As shown in Table 5 (Table 5a–Table 5i), while 55.6% of the respondents who chose Afrikaans had adequate health-related word recognition and pronunciation levels, for the same respondents only 33.3% showed adequate scores in English. For those who chose Sepedi, 65.8% showed adequate health-related word recognition and pronunciation, while in English for the same respondents only 13.9% showed adequate scores. For those who chose Sesotho as the native language for the health-related word recognition and pronunciation assessment, 90.0% showed adequate scores while in English only 21.7% showed adequate scores. For those who chose XiTsonga as the native language, 89.7% showed adequate health-related word recognition and pronunciation while only 6.9% showed adequate scores in English. For those who chose IsiZulu, 97% showed adequate health-related word recognition and pronunciation scores while 25.4% showed adequate scores in English. For those who chose Tshivenda as a native language, 95.5% showed adequate health-related word recognition and pronunciation, while in English 13.6% showed adequate scores in English. For those who chose XiTsonga as a native language, 94.3% showed adequate health-related word recognition and pronunciation, compared to 28.6% that showed adequate scores in English. For those who chose IsiZulu as a native language, 97.0% showed adequate health-related word recognition and pronunciation, compared to 25.4% that showed adequate scores in English. For those who chose Swati as a native language, 81.8% showed adequate health-related word recognition and pronunciation, compared to 18.2% who showed adequate scores in English. For those who chose Setswana, 68.8% showed adequate health-related word recognition and pronunciation compared to 17.5% who showed adequate score in English.

## Discussion

Health literacy is defined as the skills and competencies that people need to find, comprehend, evaluate, and use health information. Health literacy also involves concepts to make informed decisions to reduce health risks and improve the quality of life.^32^ In this study, we only assessed one component of HL, which is to recognise and pronounce health-related words as part of print (reading and writing).^1^ Reading and writing is the foundation of HL and therefore forms an important part of the assessment of HL.^11^ Although also important components of HL, comprehension, evaluation, and use of health information were not assessed in this study. These components rather form part of cultural and conceptual knowledge, oral HL (listening and speaking), and numeracy.^11^ Assessing PHC patient’s ability to recognise and pronounce health-related words commonly used in the PHC context remains an important aspect of HL as comprehension and evaluation are higher order skills stemming from word recognition and the ability to pronounce health-related words correctly.^12^ Improving patient’s ability to understand health information is an important aspect in empowering patients in the PHC context to take responsibility for and manage their own health, and that of their families.^32^ A low or inadequate ability to recognise and pronounce health-related words as a component of HL is associated with inadequate knowledge about health and the healthcare system, the use of health services, and increased hospitalisation.^32^ The findings of the study gave a comparison between the health-related word recognition and pronunciation levels of PHC patients in English and in the patient’s native languages.

Increased age, educational levels, poor socio-economic status, and poor English proficiency and reading levels are major barriers to components of HL.^8,28^ In our study, more than half of the respondents were between the ages of 30–40 years (53%) and 18–29 years (32%). The ability to correctly read and understand medical information declines with age.^27^ Few of the respondents were older than 50 years (14.1%). With older age, the risk for incorrect taking of medication, poor chronic disease management, low use of preventative health services, and the risk of mortality increase.^4,37,38^ Lee and Lee^4^ have reported that the components of HL levels drastically decline over the age of 40 years. The authors also mention that the level of understanding of medical text declines in older patients as compared to younger adults.^4^

It is well established that educational levels are a key determinant of health.^6^ It is also reported that a higher level of education is linked to higher total HL levels.^4,27,37^ In our study, 54% of the respondents completed a grade 12 level of schooling, while 42% did not complete high school but only completed some high school level education. It has been reported that there is a positive correlation between school grade levels and total HL levels in younger and middle-aged patients (40–65 years).^4^ However, although 54% of the respondents completed grade 12 and would be expected to be literate in English, only 19.5% of the respondents showed adequate health-related word recognition and pronunciation levels in English. These results are worrisome as health-related word recognition and pronunciation are important in the PHC context as patients often need to read prescriptions, medication labels, patient education materials, medical forms, and appointment slips for follow-up visits.^9^ Healthcare providers (HCP) often assume that when a patient is literate or come across as literate, they can understand health information and have high total HL levels.^8^ This is often not the case, as even patients with high literacy skills may have trouble recognising health-related words and understanding health information in either written or oral form as the demand in the healthcare context is often more complex than in the context of everyday life.^1^ Janse van Rensburg reported that the health-related word recognition and pronunciation as a component of HL levels of PHC patients are as much as four school grade levels below the highest level of schooling obtained.^12^ The authors also mention that some patients may also lack the ability to comprehend what they hear or read because the average person’s English comprehension level is five grades below the highest level of schooling.^12^ Considering the finding by Janse van Rensburg,^12^ none of the respondents in this study can actually be declared as health literate in the component of having the ability to adequately recognise health-related words and reading. There is a clear mismatch between the patient’s highest level of schooling, English comprehension levels, and their ability to read and pronounce health-related words.^12^ It is also reported that patients, even those with high literacy levels, are often too embarrassed to indicate that they do not understand healthcare instructions and health education given by the HCP.^12^

Although HCP assume that health information and health education is understood by patients, this is rarely the case.^12^ Health education in either oral or written form is often given at a level much higher than the patient’s understanding.^12^ The benefits of giving health information and healthcare instructions at the level of the patient’s understanding, including giving health information in the patient’s native language, includes increased patient knowledge, reduced patient anxiety, and better adherence to treatment.^12^ Low HL can be managed by closing gaps between health information and health education given, and the health information/education provider by using simplified language and giving information in the patient’s native language.^27^ In our study, English was not the native language of any the respondents. It is reported that in South Africa, English is the native language of only 8.2% of the population. Even though English is the dominant language spoken in the public sphere in South Africa, the country has 11 other official languages.^15,27^ Malik^27^ emphasises that people with low or inadequate HL often misunderstand information in the English language. Although only 19.5% of the respondents showed adequate health-related word recognition and pronunciation levels in English, the results of the levels in the respondent’s native languages were far better. Posiliti and Cilliers^38^ also reported a significant difference in English HL levels and IsiXhosa HL levels in a study conducted in South Africa. Du et al.^8^ and Hargis et al.^39^ confirm that HL levels are often lower in people whose native language is not English. Patients in the PHC context need to actively engage in the management of their health and need to be able to make healthcare decisions.^1^ To do so, information needs to be appropriate and on the patient’s level of understanding.^1^ This does not only refer to HL levels but also to giving health education in the patient’s native language and taking into consideration the patient’s cultural and social backgrounds.^1^ Inappropriate understanding of health information because of low HL levels and language barriers substantially impact patient’s health behaviours and health outcomes.^1,9^ Most HCP in PHC usually speak the local native language and understanding the language might not be a problem for most patients; however, further emphasis should be placed on determining the HL level of the patients and the level of understanding at which the health information and health education is provided.^39^

Evidence shows that poor HL is associated with more hospitalisations, greater use of emergency care, poorer overall health status, and higher mortality rates.^9^ Patients with adequate HL levels, on the other hand, were shown to be more likely to comprehend health information, identify medication names, better access to health services, and maintain good health.^8,10^

### Strengths and limitations

To the best of our knowledge, this is the first study conducted to compare health-related word recognition and pronunciation levels in both English and the native languages of PHC patients in South Africa. Access to a larger sample size of PHC patients in all nine provinces of South Africa may give a more generalised picture of the comparative results. The inclusion of elderly patients may be important for future research as in our study younger and middle aged constituted most of the respondents. The IsiNdebele language was excluded from this study as this native language is not often spoken in the Gauteng province. In future studies, this native language should also be included. The results of the study were only based on the ability to read and pronounce medical words. Future studies should also include the comprehension of the medical word in English and in the native languages. The awareness of HL and the different components of HL in HCP in the PHC context may be improved by the development of a HL awareness programme. Recommendations for future research also includes the development of culturally tailored health education materials, providing language interpretation services in PHC clinics, and implementing HL assessment tools in native languages. Exploring the effectiveness of different approaches for improving HL levels among PHC patients, evaluating the impact of culturally tailored health education interventions, and assessing the long-term effects of improving HL on patient outcomes is also recommended.

## Conclusion

Healthcare providers in the South African PHC context provide care to patients of a diverse cultural and linguistic background. For PHC patients, language specifically plays an important role in HL levels. A significant difference in the health-related word recognition and pronunciation levels in English and that in the native language were observed in this study. The significance of this is that although patients indicate that they have completed high school, this may not be a true reflection of their ability to read and understand health-related words used in the PHC setting. Providing health information and health education in the patient’s native language and on a level of the patient’s understanding may therefore improve the health outcomes of the patients and prevent referral of PHC patients to higher levels of healthcare. The complexity of the South African healthcare system and an increasing burden of disease demand a greater participation of PHC patients in self-care, further emphasising the importance of HCP’s awareness of the HL of PHC patients in the PHC context of the country.
